# Absolute reliability of Young's modulus of the soleus muscle and Achilles tendon measured using shear wave elastography in healthy young males

**DOI:** 10.1016/j.asmart.2024.04.001

**Published:** 2024-04-20

**Authors:** Hayato Miyasaka, Bungo Ebihara, Takashi Fukaya, Hirotaka Mutsuzaki

**Affiliations:** a Department of Rehabilitation, Tsuchiura Kyodo General Hospital, 4-1-1 Otsuno, Tsuchiura-shi, Ibaraki, 300-0028, Japan; b Department of Physical Therapy, Faculty of Health Sciences, Tsukuba International University, 6-8-33 Manabe, Tsuchiura-shi, Ibaraki, 300-0051, Japan; c Center for Medical Science, Ibaraki Prefectural University of Health Sciences, 4669-2 Ami, Ami-machi, Inashiki-gun, Ibaraki, 300-0394, Japan; d Department of Orthopedic Surgery, Ibaraki Prefectural University of Health Sciences Hospital, 4773 Ami, Ami-machi, Inashiki-gun, Ibaraki, 300-0331, Japan

**Keywords:** Bland–Altman plot, Minimal detectable change, Reliability, Shear wave elastography, Young's modulus

## Abstract

**Background:**

Stiffness of the soleus muscle (SOL) and Achilles tendon (AT) are associated with Achilles tendinitis and medial tibial stress syndrome. Therefore, reliable SOL and AT stiffness measurements are important for monitoring clinical progress. However, little is known about the absolute reliability of the stiffness measurements of SOL and AT in different ankle positions. This study aimed to determine the absolute reliability of the Young's modulus measurements of the SOL and AT in different ankle positions in healthy young males.

**Methods:**

This study included 33 healthy young males. SOL and AT stiffnesses were measured using Young's modulus and shear-wave elastography (SWE). Measurements were taken while the participants were kneeling, with their knees flexed to 90°, and the upper body supported by a table. Ultrasound images were recorded at ankle dorsiflexion angles of −10°, 0°, and 10°. The same measurements were repeated 15 min after the first measurement. Bland–Altman plots were used to verify the type or amount of error and 95 % confidence interval of the minimal detectable change (MDC_95_) values of the measurements.

**Results:**

Bland–Altman plots identified that there was no fixed or proportional bias and that there was good agreement between the first- and second-time measurements of the SOL and AT, respectively, among all angles. The MDC_95_ of the Young's modulus of SOL at −10°, 0°, and 10° of ankle dorsiflexion were 5.6 kPa, 7.0 kPa, and 10.1 kPa, respectively, and AT were 15.8 kPa, 16.4 kPa, and 17.8 kPa, respectively.

**Conclusion:**

Young's modulus measurements of the SOL and AT using SWE can be used to quantify elastic properties with high confidence. Clinically, assessing changes in the Young's moduli of the SOL and AT using SWE may help determine the effectiveness of interventions.

## 1 Introduction

The largest and strongest tendon in the body is the Achilles tendon (AT), which connects the soleus muscle (SOL) to the calcaneus.^1^^,^^2^ The AT is one of the most frequently injured tendons.^3^ It has been reported that a stiffer AT is a risk factor associated with Achilles tendinitis.^4^ SOL stiffness is also involved in the development of medial tibial stress syndrome (MTSS).^5^ It has also been reported that the SOL tendon is most stretched during both ankle internal and external rotation and thus is involved in the development of AT disorders.^6^ We focused on the SOL because overstrain of the SOL increases tensile stress on the AT and is thought to be a factor causing overuse syndrome.Using stretching, increasing the flexibility of the triceps surae, and improving the stiff region of the SOL and AT may prevent or improve overuse injuries. Therefore, reliable SOL and AT stiffness measurements are important for monitoring clinical progress.

Shear wave elastography (SWE) is a rapidly developing method that is useful for determining the stiffness of a range of human tissues^7^^,^^8^ and is represented in terms of the Young's modulus. Increased tissue stiffness is indicated by an increased Young's modulus. SWE can be applied both before and after stretching the soft tissues to assess the effects of stretching.^9^ However, the measurement of Young's modulus depends on the skill of the tester.^10^ Several studies have assessed the reliability of SWE measurements using measures of relative reliability, such as the intraclass correlation coefficient (ICC)^11^^,^^12^; however, this method is limited by the fact that it cannot derive the type or amount of error. Therefore, the difference in the Young's modulus before and after stretching may include random error and systematic bias, in addition to the effect of stretching.

The Bland–Altman plot is a graphical technique for finding any connections between the differences and averages of scores on two tests, a study of absolute reliability.^13^ The technique complements the minimum detectable change (MDC) in establishing the test-retest reliability of measurement methods. The MDC is one of the methods used to assess the measurement error and is the magnitude of the measurement error among the changes in two measurements obtained by repeated measurements, such as retests. The 95 % confidence interval of the MDC (MDC_95_) is generally used.^14^ Changes in measurements within the MDC_95_ are due to measurement error, whereas changes greater than the MDC_95_ are true changes and can be judged to be the effect of the intervention. Therefore, using MDC_95_, it may be possible to evaluate whether the difference in Young's modulus before and after stretching is due to the effect of stretching or a measurement error. The absolute reliability of Young's modulus measurements of the gastrocnemius has already been reported^15^; however, reports on the absolute reliability of the SOL are limited. In particular, there are no reports on the absolute reliability of Young's modulus measurements of the SOL at different ankle positions with a flexed knee using the Bland–Altman plot.

This study aimed to determine the absolute reliability of Young's modulus measurements of SOL and AT in different ankle positions in healthy males using Bland–Altman plot analysis; once the reliability and MDC_95_ of SOL and AT Young's modulus is determined, it can be used to help determine the effectiveness of an intervention.

## 2 Materials and methods

### 2.1 Study design

This cross-sectional study included 33 healthy young males. The ethics committee of our institute approved this study, which was conducted in accordance with the principles of the Declaration of Helsinki. All participants provided written informed consent before participation.

### 2.2 Participants

The study was conducted in 2023 and used hospital networks and posters to recruit healthy men from among hospital employees. Using the method of Lu M et al.,^16^ a sample size calculation was performed for the Bland–Altman plot, with standardised agreement limits of 3.0, standardised difference limits of 0, an error probability of 0.05, and a power of 0.8, and 33 healthy young males participated in the present study. Regarding physical activity level, participants engaged in light work or recreational sports.The dominant leg was assessed by asking the participants which leg they would use to kick a ball,^17^ and measurements of the right foot were performed for all participants. The participants included those who (1) had a maximum ankle dorsiflexion ROM of at least 10°, (2) had no fever, (3) had no joint pain or muscle pain, and (4) could understand and sign a consent form. Participants with a history of neuromuscular disease or musculoskeletal injury in the lower limbs were excluded.

### 2.3 Measurement of Young's modulus of SOL and AT

**Fig. 1:**
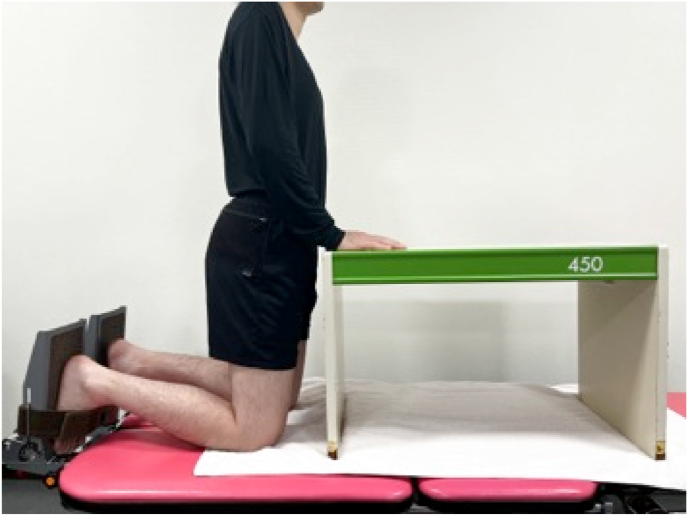
Illustration of the measurement position of Young’s modulus of soleus muscle and Achilles tendon. The participants were measured while kneeling, with their knees flexed to 90° and the upper body supported by a table.

**Fig. 2:**
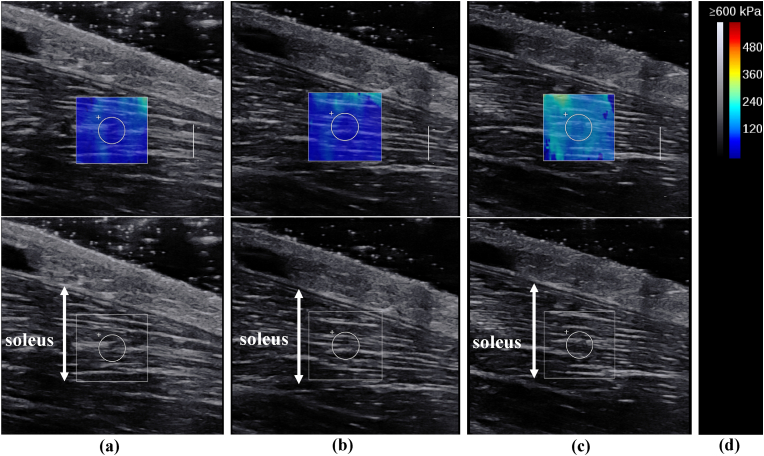
Typical example of elasticity maps of the soleus muscle during passive ankle dorsiflexion at (a) −10°, (b) 0°, and (c) 10°. The grey and colour scale (d). (For interpretation of the references to colour in this figure legend, the reader is referred to the Web version of this article.)

**Fig. 3:**
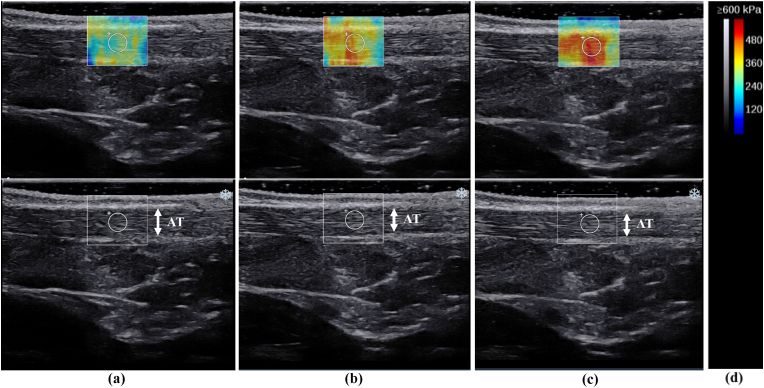
Typical example of elasticity maps of Achilles tendon during passive ankle dorsiflexion at (a) −10°, (b) 0°, and (c) 10°. The grey and colour scale (d). (For interpretation of the references to colour in this figure legend, the reader is referred to the Web version of this article.)

All ultrasound examinations were performed using a 2–10 MHz linear transducer (Supersonic Imaging, Aix-en-Provence, France). A physical therapist with six years of expertise in performing musculoskeletal ultrasound tests assessed the Young's modulus using the SWE Opt penetration mode. The Young's modulus ranges for SOL and AT were 0–600 and 0–800 kPa, respectively. The room temperature was controlled at 25 °C.^18^ The Young's modulus E was calculated as follows:

E = 3ρc^2^

where E is the Young's modulus, ρ is tissue density and c is shear wave velocity.^19^ Measurements were performed with the participants kneeling with their knees flexed to 90° with their upper bodies supported by a table. They were instructed to remain relaxed during the measurements (Fig. 1).^20^ Owing to their effect on muscle stiffness, participants were instructed to avoid exercises that could result in a delayed onset of muscle soreness for two days prior to the measurement day.^21^ Ultrasound images were recorded along the longitudinal axis of the muscle and tendon at ankle dorsiflexion of −10°, 0°, and 10°. The ankle joint was securely attached to a tilt-table footplate. The SOL regions were measured near the muscle-tendon transition part of the gastrocnemius, and the AT regions were measured 3 cm above the calcaneal tuberosity.^22^^,^^23^ These levels may be clinically important sites because they are prone to overuse injuries due to stiffness of the SOL and AT.^24^^,^^25^ In addition, the relatively superficial position allows for Young's modulus measurements.The muscle-tendon transition part was identified using the B-mode horizontal axis image. The skin surface was marked using a marker. The probe was held stationary for 10 s using region of interest (ROI) circles of 4 mm and 3 mm in diameter on the SOL and AT, respectively.^26^ The ROI was set near the centre of the SOL (Fig. 2) and AT (Fig. 3). A large amount of gel was applied with a light touch of the probe to minimise the pressure on the skin.

To verify the reliability of the measurements, the same measurements were repeated 15 min after the first measurements.^27^ Participants sat and rested between measurements.

### 2.4 Statistical analysis

The Shapiro–Wilk test was used to evaluate the distribution of the measured values. Means and standard deviations were used for distributed data, and medians and interquartile ranges were used for non-distributed data. The reliability of the measurements was evaluated using ICC and Bland–Altman plots. Using the measurement results of the Young's moduli of the SOL and AT, the intra-rater reliability was calculated as ICC (1, 1). The ICC (1, 1) was calculated along with the standard error of the mean (SEM),^28^ MDC_95_ values, and relative repeatability (RR)^29^ to determine intra-rater reliability. Bland–Altman analysis and ICC were assessed from the average value of the Young's modulus of the first and second measurements. The SEM was calculated by the formula SEM = standard deviation × √(1 − ICC), the MDC_95_ was computed by the formula MDC_95_ = 1.96 × SEM × √2, and the RR was calculated by the formula RR = MDC_95_/mean. Ankle position effects were shown using one-way repeated measurements, analysis of variance, and the Bonferroni post hoc test. Statistical significance was set at p < 0.05. Modified R commander version 2.8.1 (CRAN, Freeware) was used to perform all statistical analyses.

## 3 Results

### 3.1 Participants’ characteristics

The participants’ characteristics are summarised in Table 1. The median age of the participants was 27.0 years (interquartile range: 25.0–34.0).

**Table 1:** Physical characteristics of the participants.

Age (years)	27.0 (25.0–34.0)
Height (m)	1.72 (1.70–1.75)
Weight (kg)	63.6 (61.8–70.0)
Body mass index (kg/m^2^)	21.5 (20.7–22.7)
Dominant leg (right/left)	31/2

Values are presented as medians (interquartile ranges) or *n*/*n*.

### 3.2 Intra-rater reliability of the Young's modulus of SOL and AT

**Fig. 4:**
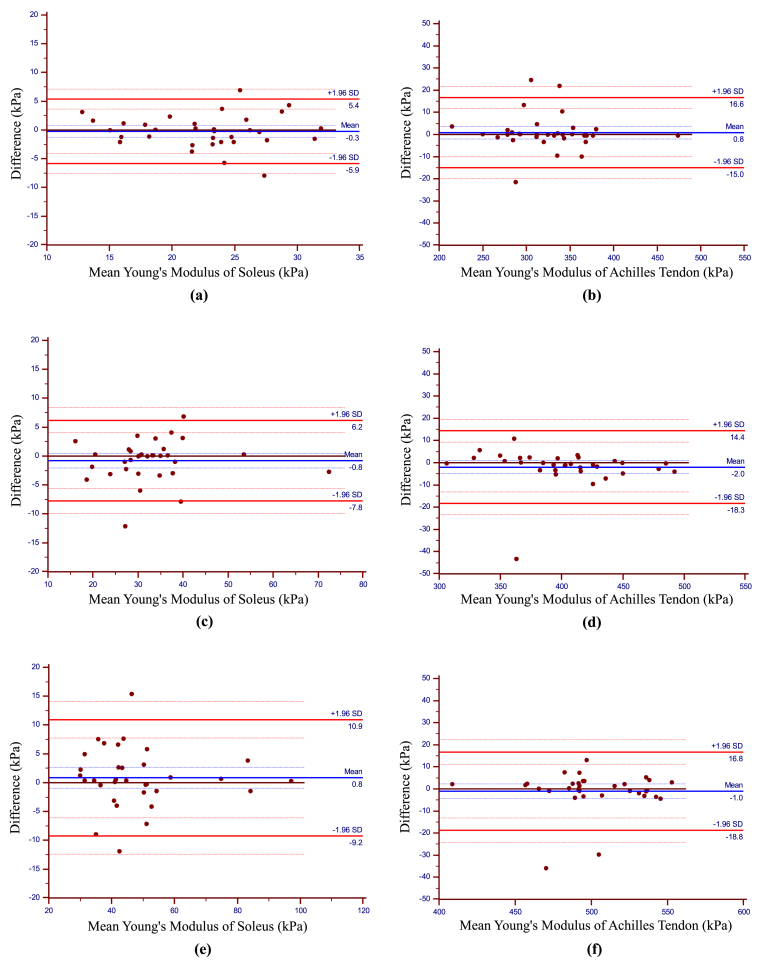
Bland–Altman plots of intra-rater reliability of the Young's Modulus of Soleus muscle (a: 10°; c: 0°; e: 10°) and Achilles tendon (b: 10°; d: 0°; f: 10°). The differences in Young's modulus between the first and second measurements are plotted against the mean of each participant for the soleus muscle and Achilles tendons. The blue line represents the mean of the differences, and the red line indicates the limits of agreement from −1.96 standard deviations to +1.96 standard deviations. The blue dotted line and red dotted line indicate the 95 % confidence interval of the mean difference and the 95 % confidence interval of ±1.96 standard deviations, respectively.

The Bland–Altman plots of the intra-rater reliability values are shown in Fig. 4. The mean differences of the Young's Modulus of SOL at −10°, 0°, and 10° of ankle dorsiflexion were −0.3 kPa, −0.8 kPa, and 0.8 kPa, respectively, and those of AT were 0.8 kPa, −2.0 kPa, and −1.0 kPa, respectively. There was no fixed or proportional bias, and there was good agreement between the first and second measurements for all SOL and AT angles. The ICC, SEM, MDC_95_, and RR for the intra-rater reliability of the Young's Modulus of SOL and AT are shown in Table 2. The ICC (1.1) values of the Young's modulus of SOL at −10°, 0°, and 10° of ankle dorsiflexion were 0.85, 0.94, and 0.95, respectively, and those of AT were 0.99, 0.98, and 0.96, respectively. The SEM of the Young's modulus of SOL at −10°, 0°, and 10° of ankle dorsiflexion were 2.02 kPa, 2.52 kPa, and 3.63 kPa, respectively, and those of AT were 5.70 kPa, 5.90 kPa, and 6.41 kPa, respectively. The MDC_95_ of the Young's modulus of SOL at −10°, 0°, and 10° of ankle dorsiflexion were 5.6 kPa, 7.0 kPa, and 10.1 kPa, respectively, and that of AT was 15.8 kPa, 16.4 kPa, and 17.8 kPa, respectively. The RR of the Young's modulus of SOL at −10°, 0°, and 10° of ankle dorsiflexion were 0.25, 0.21, and 0.21, respectively, and that of AT was 0.05, 0.04, and 0.04, respectively.

**Table 2:** Intra-rater Reliabilities of Shear Wave Elastography for the Young's Modulus of the Soleus muscle and Achilles tendon.

Measurement tissue	Ankle position	Test 1 (kPa)	Test 2 (kPa)	ICC	95 % CI	SEM (kPa)	MDC_95_ (kPa)	RR
Soleus muscle	−10°	22.5	22.8	0.85	0.72–0.92	2.02	5.6	0.25
	0°	32.4	33.2	0.94	0.88–0.97	2.52	7.0	0.21
	10°	48.4	47.6	0.95	0.90–0.97	3.63	10.1	0.21
Achilles tendon	−10°	324.4	323.6	0.99	0.97–0.99	5.70	15.8	0.05
	0°	400.2	402.2	0.98	0.96–0.99	5.90	16.4	0.04
	10°	501.0	502.0	0.96	0.92–0.98	6.41	17.8	0.04

ICC, intraclass correlation coefficient; 95 % CI, 95 % confidence interval; SEM, standard error of the mean; MDC_95_, 95 % confidence interval of the minimum detectable change; RR, relative repeatability.

### 3.3 Young's modulus of SOL and at in −10°, 0°, and 10° of ankle dorsiflexion

**Fig. 5:**
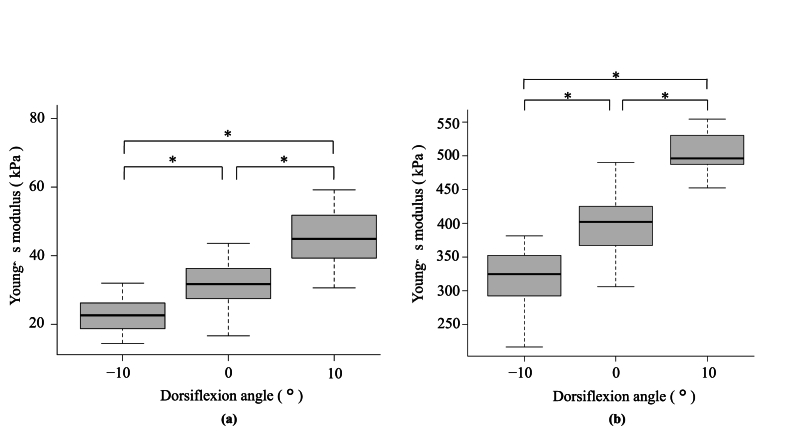
Comparison of Young's modulus of the soleus muscle (a) and Achilles tendon (b) at −10°, 0°, and 10° of ankle dorsiflexion. * One-way repeated-measures analysis of variance and Bonferroni post-hoc test detected significant differences among all angles (p < 0.001). The mean values and the 25th and 75th percentiles are indicated by black lines inside the boxes and at the boundaries of the boxes.

The differences in the Young's modulus between the ankle positions are summarised in Fig. 5. The mean Young's modulus of the SOL at −10°, 0°, and 10° of ankle dorsiflexion were 22.5 ± 5.1°, 32.4 ± 10.5°, and 48.4 ± 16.1°, respectively, and that of AT was 324.4 ± 47.2°, 400.2 ± 44.1°, and 501.0 ± 31.9°, respectively.

## 4 Discussion

This study estimated the absolute reliability of Young's modulus measurements of the SOL and AT in different ankle positions in the flexed knee using Bland–Altman plots and calculated the MDC_95_. The results showed that the measurement of the Young's modulus of the SOL and AT using SWE had excellent reliability, and the relatively low values of MDC_95_, SEM, and RR supported the accuracy of the assessment. In addition, visual inspection of the Bland–Altman plots showed no signs of systematic bias. Therefore, the MDC_95_ might be considered the optimum criterion for determining whether changes in the Young's modulus of the SOL and AT are real changes or the result of random error.

It has been reported that Young's modulus of SOL and AT measured with the extended knee position in the prone position is highly reliable if the ankle is dorsiflexed.^30^^,^^31^ SOL is the uniarticular plantar flexor muscle, and measuring Young's modulus in the knee flexion position may better reflect the influence of the SOL.^32^ The strength of the reliability coefficient is defined as excellent (>0.90), good (0.71–0.90), moderate (0.50–0.70), or poor (<0.50).^33^ This study demonstrated good to excellent reliability for all ankle angles.

The MDC_95_ was computed to provide values that reflected true differences that exceeded the measurement error. The result showed that the MDC_95_ of the Young's modulus of SOL at −10°, 0°, and 10° of ankle dorsiflexion were 5.6 kPa, 7.0 kPa, and 10.1 kPa, respectively, and AT were 15.8 kPa, 16.4 kPa, and 17.8 kPa, respectively. Therefore, the Young's moduli of the SOL and AT should be greater than these values to reflect the effect of the intervention. Furthermore, as the ankle dorsiflexion angle increased, the MDC_95_ values of Young's modulus increased. Therefore, the greater the ankle dorsiflexion angle, the more caution is required to interpret the results of the Young's modulus.

Moreover, Young's moduli of the SOL and AT increased as the ankle was dorsiflexed. The SOL operates on the plantar flexion of the ankle via the AT.^34^ Young's modulus may have increased as a result of passive tension generated by stretching the SOL and AT with ankle dorsiflexion.

Clinically, assessing changes in Young's moduli of the SOL and AT using SWE may help determine the effects of interventions and changes over time. This method can be used to measure various ankle angles and may also be useful for patients with limited ankle range of motion.

This study had several limitations. First, the inter-rater reliability was not examined. Therefore, we were unable to identify whether the reliability-measured Young's moduli of the SOL and AT were comparable across testers. Second, electromyography was not used during the SWE measurements. To ensure that there is no muscular contraction, it is vital to monitor muscle activity. Third, physical activity may affect the mechanical and morphological properties of the AT.^35^ Finally, the study participants were slender, healthy, young males. Whether participants of different ages, sexes, and body mass indices would respond differently remains unknown.In addition, these results may be of limited value in studies investigating patients with tendinopathy.Further studies are required to overcome these limitations.

## 5 Conclusion

Young's modulus measurements of the SOL and AT using SWE can be used to quantify the elastic properties with high confidence. To reflect the effect of the intervention, the Young's moduli of the SOL and AT must change more than the MDC_95_ values shown here.

## Conflicts of interest

The authors declare no conflict of interest.

## Funding statement

This research did not receive any specific grant from funding agencies in the public, commercial, or not-for-profit sectors, and no material support of any kind was received.

## Ethics approval and consent to participate

This study was approved by the ethics committee of Tsuchiura Kyodo General Hospital (Reference number: 2023FY121). Written informed consent was obtained from all the participants.

## Funding

This research received no external funding.

## Availability of data and materials

The data that support the findings of this study are available on request from the corresponding author. The data are not publicly available, owing to restrictions on their containing information that could compromise the privacy of research participants.

## Declaration of competing interest

The authors declare no conflict of interest.
